# Enhanced Mucoadhesion of Thiolated β-Cyclodextrin by S-Protection with 2-Mercaptoethanesulfonic Acid

**DOI:** 10.1021/acsomega.3c08836

**Published:** 2024-01-26

**Authors:** Gergely Kali, Ali Magdi Mahmoud Mahmoud Taha, Emiliano Campanella, Martyna Truszkowska, Soheil Haddadzadegan, Nunzio Denora, Andreas Bernkop-Schnürch

**Affiliations:** † Center for Chemistry and Biomedicine, Department of Pharmaceutical Technology, Institute of Pharmacy, University of Innsbruck, Innrain 80-82, A-6020 Innsbruck, Austria; ‡ Department of Pharmacy, University of Bari Aldo Moro, Piazza Cesare Battisti, I-70121 Bari, Italy

## Abstract

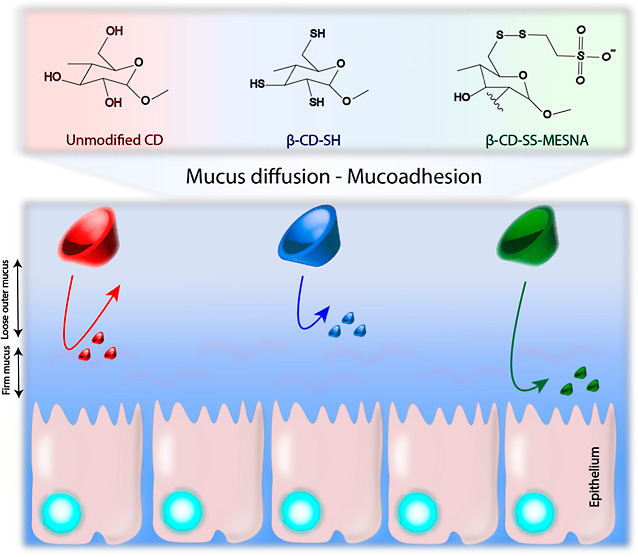

This study aimed at designing an S-protected thiolated β-cyclodextrin (β-CD) exhibiting enhanced mucoadhesive properties. The native β-CD was thiolated with phosphorus pentasulfide resulting in a thiolated β-CD (β-CD-SH) and subsequently S-protected with 2-mercaptoethanesulfonate (MESNA) to form β-CD-SS-MESNA. The structure of the novel excipient was confirmed by ^1^H NMR and Fourier-transform infrared spectroscopy. The sulfhydryl content of β-CD-SH, determined by Ellman’s test, was 2281.00 ± 147 μmol/g, and it was decreased to 45.93 ± 19.40 μmol/g by S-protection. Due to thiolation and S-protection, the viscosity of the mixture of mucus with β-CD-SH and β-CD-SS-MESNA increased 1.8 and 4.1-fold, compared to native β-CD, respectively. The unprotected β-CD-SH diffused to a lesser extent into the mucus than native β-CD, while S-protected β-CD-SS-MESNA showed the highest mucodiffusion among the applied CDs. A 1.5- and 3.0-fold higher cellular uptake of β-CD-SH and β-CD-SS-MESNA, compared to the native one, was established on Caco-2 cell line by flow cytometry, respectively, causing slightly decreased cell viability. On account of the enhanced mucoadhesion, this higher cellular uptake does not affect the application potential of β-CD-SS-MESNA as an oral drug delivery system since the carrier remains in the mucus and does not reach the underlying epithelial layer. According to these results, the S-protection of β-CD-SH with MESNA promotes improved mucodiffusion, strong mucoadhesion, and prolonged mucosal residence time.

## Introduction

Cyclodextrins (CDs) are ring-shaped oligosaccharides that are used in numerous drug delivery systems. They are utilized to complex, solubilize, and protect drugs in an aqueous biological environment.^1,2^ One of the key advantages of CDs is their small size with a height and external diameter of up to 7.9 and 16.9 Å, respectively, making them likely the smallest drug carrier systems.^3,4^ One limitation of CDs as drug delivery systems is that they do not interact with the mucus layer, resulting in a short mucosal residence time.^5^ Mucoadhesion is provided by ionic interactions and hydrogen bonding in combination with the interpenetration of polymeric chains into the mucus gel layer, followed by chain entanglements.^6−8^ Since CDs are nonionic and too small for chain entanglements, they are not mucoadhesive at all. To trigger prolonged mucosal residence time, modification of CDs with thiol groups is in focus of recent research.^9−15^ The sulfhydryl groups can form disulfide bonds with cysteine moieties of mucus glycoproteins and immobilize the carrier in the mucus layer.^16^ Thiolated CDs are investigated mostly for ocular and oral drug delivery.^13,14,17^ They can be generated by covalent attachment of thiol-containing ligands^18−20^ or direct hydroxyl-to-thiol conversions.^10−12,14,21,22^ The latter is preferred because it does not alter the CDs’ characteristics, such as its complexation ability or cytotoxicity.^9^ A crucial factor for mucoadhesion is the degree of thiolation, as the more the hydroxyls exchanged with thiol groups, the stronger mucoadhesion.^9,11,14^ Recently, our research group described a thiolation process, applying phosphorus pentasulfide, and reached an up to 100% degree of thiolation.^10^ This per-thiolated β-cyclodextrin (β-CD) presented up to 89-fold enhancement in mucoadhesive properties in vitro and also highly prolonged gastrointestinal residence time in vivo.^10,23^ Despite these advantageous mucoadhesive properties, the aqueous solubility of thiolated β-CDs is similar or lower than that of the native CD. This poor solubility could be addressed by the use of other, more soluble CD derivatives, such as methylated or hydroxypropylated β-CDs.^13,15^

The main limitation of thiolated drug delivery systems, besides the limited aqueous solubility, is that the thiol groups tend to oxidize to disulfides, lowering the available free thiol content. Moreover, mucoadhesion occurs already on the outer, loose mucus layer that is rapidly eliminated by the mucus turnover process, strongly limiting the residence time of the thiolated drug carriers. This shortcoming can be addressed by less reactive, S-protected thiolated polymers and oligomers.^24^ These protected thiomers are stable against oxidation and can diffuse into the deep firm mucus layer to a greater extent before being immobilized via oxidative disulfide bond formation. For this new disulfide bond formation, S-protected thiomers undergo a displacement mechanism. In detail, the protective group is released, and the thiol groups of the thiomer become available for disulfide bond formation with cysteine moieties of mucus glycoproteins. This interaction results in the formation of robust disulfide bonds between the thiolated CD and the mucus layer. Contrary to deactivation, the S-protecting groups serve to preactivate the free thiol moieties of the thiomer. Recent studies on S-protected thiolated polymers and CDs showed promising results of deeper mucus diffusion and enhanced mucoadhesion, up to 16-fold higher compared to native CDs.^24,25^ Mostly, 2-mercaptonicotinic acid is used for protection, but more recently, S-protection with short polyethylene glycol (PEG) chains also showed potential, as its mucodiffusion is highly supported by the mucopenetrating PEG decoration.^25^ This kind of S-protection provided an increased water solubility and improved mucodiffusive properties of the thiolated CDs.

Encouraged by these results and being aware that PEG chains can cause chain entanglements with mucus glycoproteins still limiting the mucodiffusive properties, this study aimed at designing a thiolated CD with an even more mucoinert ligand for S-protection. For S-protection of thiolated β-CD, sodium 2-mercaptoethanesulfonate (MESNA) was chosen since it is a small molecule that cannot cause chain entanglements with mucus, and it is even known for its mucolytic properties.^26^ The thiolated and S-protected CDs were characterized in terms of structure, solubility, and cell toxicity. Mucoadhesive properties and mucodiffusion were determined using porcine intestinal mucosa.

## Experimental Section

### Materials

Beta-cyclodextrin (β-CD) was purchased from Cyclolab, Hungary. The thiolating agent, phosphorus pentasulfide (P_4_S_10_, 99%); the reaction solvent, tetramethylene sulfone (sulfolane, 99%); the NMR solvent, hexadeuterodimethyl sulfoxide (DMSO-*d*_6_, 99.9%); and the model dye, 3-(2-benzothiazolyl)-7-(diethylamino)-coumarin (coumarin 6, 98%) were all ordered from Sigma-Aldrich, Austria, and used without purification. 2-Mercaptoethanesulfonate (MESNA, 98%) was obtained from Fisher Scientific, Austria. 1,4-Dithioerythritol (99%) was received from Carl Roth GmbH & Co. KG, Germany. Ellman’s reagent (5,5′-dithiobis(2-nitrobenzoic acid)), minimum essential eagle medium (MEM), and Triton X-100 were obtained from Merck, Germany, and were used as received.

### Synthesis and Purification of Thiolated CDs

The thiolated CD was synthesized according to a previously published method.^6,9^ Native β-CD (0.5 g, 0.44 mmol) and P_4_S_10_ (2.4 g, 5.3 mmol) were weighted in a 50 mL round-bottom flask and dissolved in 15 mL of sulfolane. The base, triethylamine (1 mL, 13.5 mmol), was added to the solution, purged with N_2_, heated to 130 °C, and stirred for 2 h under an inert atmosphere. Afterward, the temperature was reduced to 80 °C, and demineralized water was added slowly. The suspension was centrifuged at 2 °C and 12,500 rpm, and the precipitate was washed with ice-cold water. Finally, the product was dried until constant weight.

^1^H NMR (DMSO-*d*_6_, 400 MHz) δ/ppm = 5.69 (broad s, OH) 4.85 (s, H-1), 3.80–2.90 (m, 6H, H-2–H-6), 2.07–1.18 (s, SH).

### S-Protection of Thiolated CDs

The MESNA S-protected thiolated β-CD was synthesized in two steps. First, the MESNA dimer was prepared by oxidation of the MESNA using hydrogen peroxide in an aqueous solution at a neutral pH. MESNA (4 g) was dissolved in 50 mL of demineralized water, and the pH of the solution was adjusted to 7. Afterward, 5.3 mL of H_2_O_2_ (30%, w/v) was added dropwise, and the pH was maintained at around 8. This solution was then stirred at room temperature for 1 h and diluted to a final volume of 100 mL with demineralized water.

0.25 g of thiolated β-CD was dissolved in 25 mL of demineralized water, and 0.5 mL of 4% (m/v) aqueous MESNA dimer solution was added dropwise. The pH of the reaction mixture was adjusted to around 7, and the mixture was stirred overnight. The solution was dialyzed using a Spectrum Laboratories Biotech CE dialysis membrane (MWCO: 0.1–0.5 kDa) for 3 days, and the dialyzed product was freeze-dried.

^1^H NMR (DMSO-*d*_6_, 400 MHz) δ/ppm = 5.69 (broad s, OH) 4.85 (s, H-1), 3.80–2.90 (m, 6H, H-2–H-6), 2.86–2.71 ppm (m, -C*H*_*2*_-, MESNA).

### Characterization of Thiolated and S-Protected CDs

The sulfhydryl contents of the thiolated and S-protected thiolated CDs were quantified using Ellman’s test.^5−11^ In brief, 1 mg of the sample was dissolved in 250 μL of Ellman’s buffer, 500 μL of Ellman’s reagent (0.33 mg/mL) was added, and the resulting solution was incubated for 2 h in the dark. After centrifugation at 13,400 rpm for 5 min (MiniSpin, Eppendorf AG, Hamburg, Germany), 100 μL was transferred to a transparent 96-well plate, and absorption of this solution was measured at 450 nm using a microplate reader (Tecan Spark, Tecan Trading AG, Switzerland). The amount of sulfhydryl groups was calculated using a calibration curve of the l-cysteine solution, prepared under the same conditions. The procedure described above was used to determine the disulfide content after reducing disulfide bonds with sodium borohydride. The experiments were performed in triplicate.

^1^H NMR measurements were performed on a “Mars” 400 MHz Avance 4 Neo spectrometer from Bruker Corp. (Billerica, MA, USA, 400 MHz) in DMSO-d6 solution.

The Fourier-transform infrared (FTIR) spectra of native β-CD, β-CD-SH, and β-CD-SS-MESNA were recorded by using a Bruker ALPHA FT-IR device equipped with a Platinum attenuated total reflection module.

### Solubility Studies

From the thiolated and MESNA S-protected samples, 10 mg of the sample was incubated at 25 °C with 0.5 mL of demineralized water, and the pH of the solution was set to 7.2. After 30 min, samples were centrifuged at 13,400 rpm for 5 min (MiniSpin, Eppendorf AG, Hamburg, Germany), and 0.3 mL of supernatant was withdrawn and lyophilized (Freeze-Dryer Christ, Gamma 1–16 LSC, Germany). The dissolved amount of the modified CD was quantified gravimetrically.

The p*K*_a_ values of the thiolated CD were calculated by Chemaxon (Chemaxon, Budapest, Hungary) and Epik (Schördinger, New York, USA) protonation calculators.

### Evaluation of Cytotoxicity

#### Resazurin Assay

The cytotoxicity of the synthesized CDs cell viability studies using resazurin assay on a Caco-2 cell line were conducted.^5,6^ In brief, Caco-2 cells were seeded in 96-well plates (2.5 × 10^4^ cells per well) in penicillin/streptomycin (100 units/0.1 mg/L) and 10% (v/v) fetal calf serum containing MEM. The cell plates were incubated for 6 days at 37 °C under 5% CO_2_ and 95% relative humidity. During this incubation period, the medium was replaced every second day. Test solutions of native β-CD, β-CD-SH, and β-CD-SS-MESNA were prepared in the concentration of 0.25% (m/v) in 25 mM HEPES-glucose buffer with pH 7.4. For the experiment, cells were washed twice with buffer at 37 °C. Afterward, 0.1 mL of test solutions of the CDs, buffer as the positive control, and 0.15% Triton X-100 as the negative control were added to the cell culture plate and incubated at 37 °C in a 5% CO_2_ and a 95% relative humidity environment for 4 and 24 h. After incubation, solutions were aspirated, and cells were washed three times with preheated phosphate-buffered saline and further incubated with 150 μL of resazurin (44 μM) solution (1:20 v/v) for 2 h. The fluorescence of the supernatants was measured at 540 nm excitation and 590 nm emission wavelengths (Tecan Spark, Tecan Trading AG, Switzerland). Cell viabilities were calculated by referring to the following equation
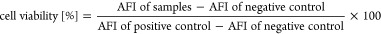
where AFI is the average fluorescence intensity. The experiments were performed in triplicate.

#### Flow Cytometry

Flow cytometry was measured as previously reported by Kaplan et al.^33^ Caco-2 cells were seeded in a 24-well plate and incubated for 14 days at 37 °C under 5% CO_2_ and a 95% relative humidity environment. Complexes of coumarin-6 and native β-CD, β-CD-SH, or β-CD-SS-MESNA (0.05% m/v) dissolved in HEPES buffer were applied to the cells and incubated for 3 h at 4 or 37 °C. Afterward, the cells were detached from the wells using trypsin (Merck KGaA, Darmstadt, Germany), followed by washing with cold phosphate-buffered saline thrice. The total population analyzed by flow cytometry (Attune NxT Flow cytometer, Thermo Fisher Scientific, MA, USA) comprised live single cells. The fluorescence of the surface-absorbed native β-CD, β-CD-SH, and β-CD-SS-MESNA coumarin-6 complexes was quenched with trypan blue (Thermo Fisher Scientific).^27^ The mean fluorescence intensity values (MFI) were used to calculate the relative mean fluorescence intensity (RMFI) values using the following formula.^28^
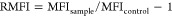


#### Life Cell Imaging Using Confocal Laser Microscopy

Confocal laser scanning microscopy (Leica TCS SP8) was employed to explore further the cellular internalization capability of β-CDs by utilizing suitable filter sets. To elaborate, Caco-2 cells were seeded in an eight-well chamber (μ-slide, Ibidi) at a density of 1 × 105 cells/mL (3 × 104 cells/well). Once confluence was achieved, cells were exposed to 0.025% labeled β-CD, β-CD-SH, and β-CD-SS-MESNA dissolved in Opti-MEM for a 3 h incubation period. Following this, the cells were washed three times with the prewarmed medium.

For nuclear staining, NucSpot Live 650 was applied according to the provider’s recommendations (1 μL of NucSpot Live 650 1000× in DMSO diluted in 1 mL of OptiMEM) for 1.5 h. Notably, as the dye exhibited nontoxicity to cells, the washing step after staining was omitted. All fluorescence images were captured under consistent conditions. Image postprocessing was conducted using ImageJ software, including *yz*- and *xz*-projections generated from 5 XY images within an image stack with a 0.2 μm *z*-step length. Spectral unmixing was employed to eliminate fluorescence bleed resulting from overlapping emission spectra in the detection channels. Additionally, 2D image filtering was controlled using a Gaussian filter.

#### Rheological Investigations

Dynamic viscosity (η), elastic modulus (*G*′), and viscous modulus (*G*″) were determined utilizing a cone–plate combination rheometer (Haake Mars Rheometer, 40/60, Thermo Electron GmbH, Karlsruhe, Germany; Rotor: C35/1°, *D* = 35 mm).^5,6^

Measurements were performed at a constant temperature of 37 °C, with a gap between the cone and the plate of 0.052 mm.

For the experiments, freshly excised porcine small intestinal mucosa, gifted from a local slaughterhouse, was cut longitudinally, and porcine mucus was collected by scrapping it off from the underlying tissue. The mucus was purified by stirring 1 g of mucus with 5 mL of 0.1 M sodium chloride solution for 1 h at 4 °C, followed by centrifugation at 10,400*g* for 2 h at 10 °C. Afterward, the supernatant was discarded, and the procedure was repeated to obtain the purified mucus. The samples were stored at −20 °C until use.

For the rheological measurement, 0.3% (m/v) native β-CD, β-CD-SH, and β-CD-SS-MESNA in 0.1 M phosphate buffer pH 6.8 were homogenized with porcine mucus in a ratio of 1:5 (v/m). After 3 h of incubation at 37 °C, samples were analyzed to determine their viscoelastic properties.

#### Preparation of Inclusion Complexes of Coumarin-6

To evaluate the cellular uptake and mucoadhesive properties of thiolated CDs, coumarin-6, a lipophilic fluorescent dye, was host–guest complexed with native β-CD, β-CD-SH, and β-CD-SS-MESNA following a previously described method.^6^ Briefly, 1 mL of ethanolic coumarin-6 solution (0.02% m/V) was added to 50 mL of 100 mM phosphate buffer pH 6.5 containing 50 mg of CD. The dispersions were stirred in the dark for 24 h at room temperature, filtered to eliminate the free dye and nondissolved CDs, and lyophilized for 2 days. The formed complexes were characterized based on their fluorescence in 100 mM phosphate buffer (pH 6.8) and ethanol, as described previously.^10^

#### In Vitro Mucoadhesion Studies on Porcine Small Intestinal Mucosa

Mucoadhesive properties of CDs were evaluated on freshly collected porcine small intestinal mucosa.^5,6^ Approximately 3 cm × 2 cm intestinal mucosa samples were glued on half-cut 50 mL falcon tubes and fixed at an angle of 45°. These tubes were placed in a thermostatic chamber (Heratherm Oven, Thermofisher Scientific, Dreieich, Germany) at 37 °C and 100% relative humidity. First, the mucosal surfaces were rinsed with 0.1 M phosphate buffer pH 6.8 for 10 min, with a flow rate of 1 mL/min using a peristaltic pump (Ismatec, IPC, High Precision Multichannel Dispenser, Richmond Scientific, Lancashire, Great Britain). In the following, 5 mg of CD/coumarin-6 host–guest complexes was placed on the mucosa and incubated for 5 min. Then, the mucosa was continuously rinsed with the same buffer with a flow rate of 1 mL/min, and samples were collected every 30 min up to 3 h. For each CD sample, 5 mg/30 mL of the CD/coumarin-6 host–guest complex in buffer, collected from the mucosa, was used as control. The samples were centrifuged at 13,400 rpm for 10 min (MiniSpin, Eppendorf AG, Hamburg, Germany), and fluorescence intensities were measured at an excitation wavelength of 480 nm and an emission wavelength of 520 nm (Tecan Spark, Tecan Trading AG, Switzerland).^5,6^

#### Mucus Diffusion Studies

The diffusion of coumarin-6, complexed in native β-CD, β-CD-SH, and β-CD-SS-MESNA into purified porcine intestinal mucus, was studied via the rotating tube method, as described previously.^15,24^ In brief, silicone tubes were cut into 50 mm long pieces and filled with 250 μL of purified porcine intestinal mucus. Afterward, 50 μL of 1% (m/v) complexes in 50 mM phosphate buffer pH 6.8 was added to the mucus-filled tubes. These tubes were subsequently closed and rotated in an incubator at 37 °C in the dark for 24 h. Thereafter, the tubes were frozen at −80 °C and cut into 8 slices of lengths of 2 mm. Each test slice was stirred with 200 μL of 96% ethanol for 3 h and centrifuged for 5 min at 13,400 rpm with a MiniSpin Centrifuge (Eppendorf, Hamburg, Germany). Fluorescence intensity was measured at 445 nm excitation and 510 nm emission wavelengths (Tecan Spark, Tecan Trading AG, Switzerland). The percentage of the polymer diffused into the mucus was calculated based on a calibration curve.^12,15^

#### Statistical Data Analysis

Statistical analyses of all data were implemented using Student’s *t*-test, with a confidence interval (CI) of *p* < 0.05. One-way analysis of variance was employed to compare data groups with 95% CI. Results were illustrated as means of at least triplicates ± SD.

## Results and Discussion

**Figure 1:**
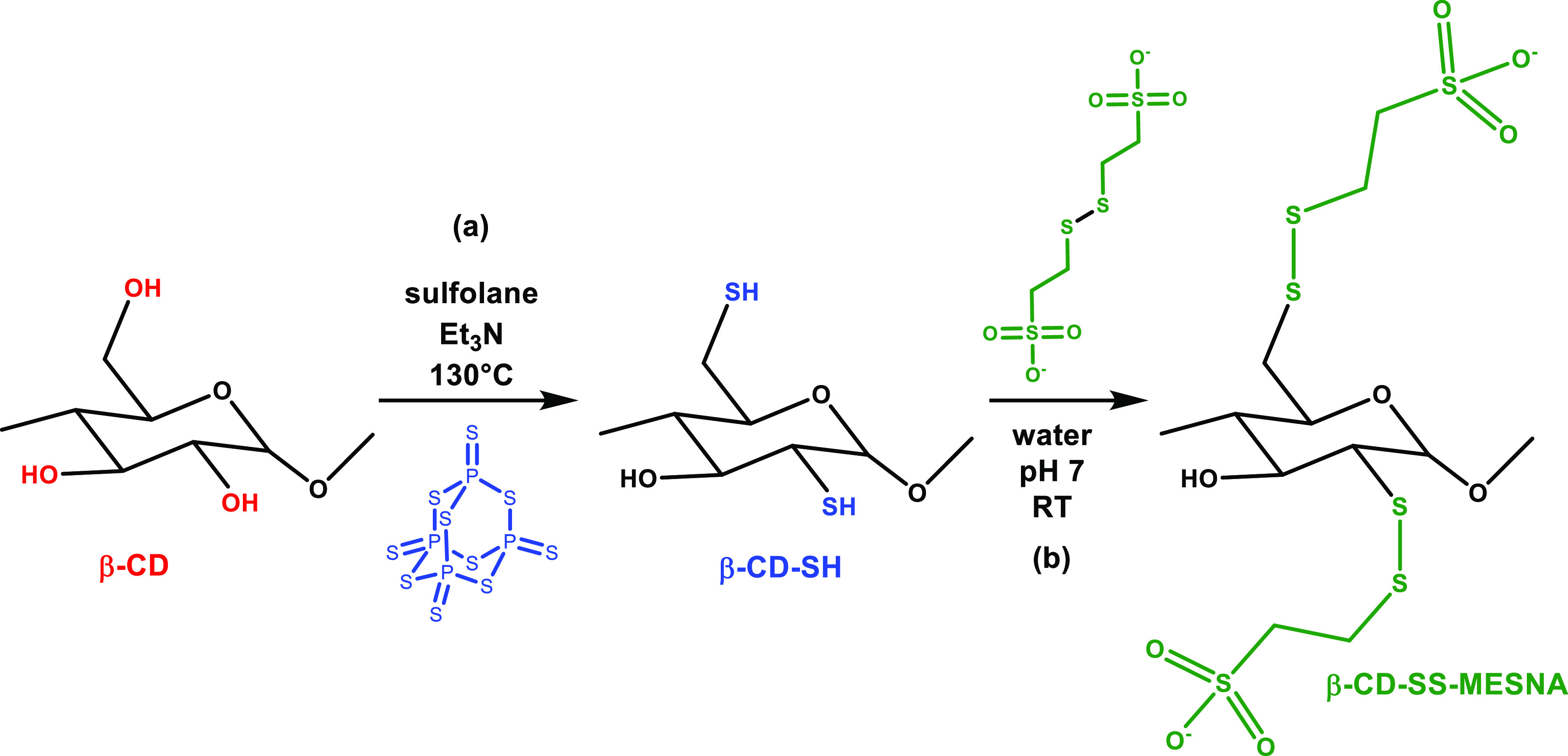
Schematic representation of the thiolation of the β-CD (a) and its S-protection with MESNA (b).

Using our previously established method, the β-CD was thiolated via the direct conversion of hydroxyl to thiol.^10^ For this, the native β-CD was reacted with P_4_S_10_ in sulfolane in the presence of Et_3_N (Figure 1a). S-protected β-CD-SS-MESNA was formed via a thiol/disulfide exchange reaction between the MESNA dimer and β-CD-SH (Figure 1b).

Due to the reduced peaks of the hydroxyl groups at the C-2 and 3, as well as C-6 positions at 5.90 and 4.20 ppm in the ^1^H NMR spectrum (Figure S1 in the Supporting Information) of the thiolated product and the new peaks for sulfhydryl groups at 2.05 and 1.15 ppm, respectively, the successful formation of β-CD-SH could be confirmed with around two thiol groups per anhydroglucose repeat. Most likely, the hydroxyl groups at C-6 and C-2 position are derivatized due to the sterical hindrance of C-3 hydroxyls. FTIR spectroscopy also confirmed the thiolation due to the reduced intensity of the −O–H stretching peak at 3200–2700 cm^–1^, while the −S–H stretching vibrations above 2500 cm^–1^ appeared (Figure S2 in Supporting Information). These vibrations from the sulfhydryl functionalities disappeared after further modification of the β-CD-SH with MESNA. Also, in the ^1^H NMR spectrum of this S-protected thiolated CD, novel peaks at 2.86–2.65 ppm appeared belonging to the methylene protons in the MESNA substructure.

The amount of free thiols and disulfide bonds on the thiolated and S-protected CDs was determined via Ellman’s and disulfide bond tests, respectively. In the case of β-CD-SH, the concentration of free thiols was 2281.00 ± 147 μmol/g, while 539.60 ± 193 μmol/g of disulfide bonds were found. After the reaction with MESNA, the amount of free thiols dropped to 45.93 ± 19.40 μmol/g, while a high quantity of disulfide bonds was detected (2735.80 ± 307.49 μmol/g), confirming the success of S-protection.

The solubility of the β-CD-SH and β-CD-SS-MESNA was investigated in demineralized water according to OECD guidelines (OECD, 1995) at pH 7.2.^29^ The results showed that β-CD-SH has a low aqueous solubility of 6.35 ± 1.16 mg/mL that increases 2.25-fold to 14.25 ± 0.34 mg/mL after S-protection. The p*K*_a_ values of the thiol groups on β-CD-SH, calculated using Chemaxon and Epik p*K*_a_ calculating tools, are around 8.3 and 7.2 for positions C6 and C2, respectively. The experimentally determined p*K*_a_ values for thiolated β-CDs are also in this range, around 8.2, close to cysteine.^9^ This p*K*_a_ value indicates low thiolate anion formation at the given pH and, consequently, low aqueous solubility of the synthesized β-CD-SH. The increased solubility in water in the case of β-CD-SS-MESNA is likely due to the anionic sulfonate moieties in the protecting groups.

### Evaluation of Cytotoxicity by Resazurin Assay

**Figure 2:**
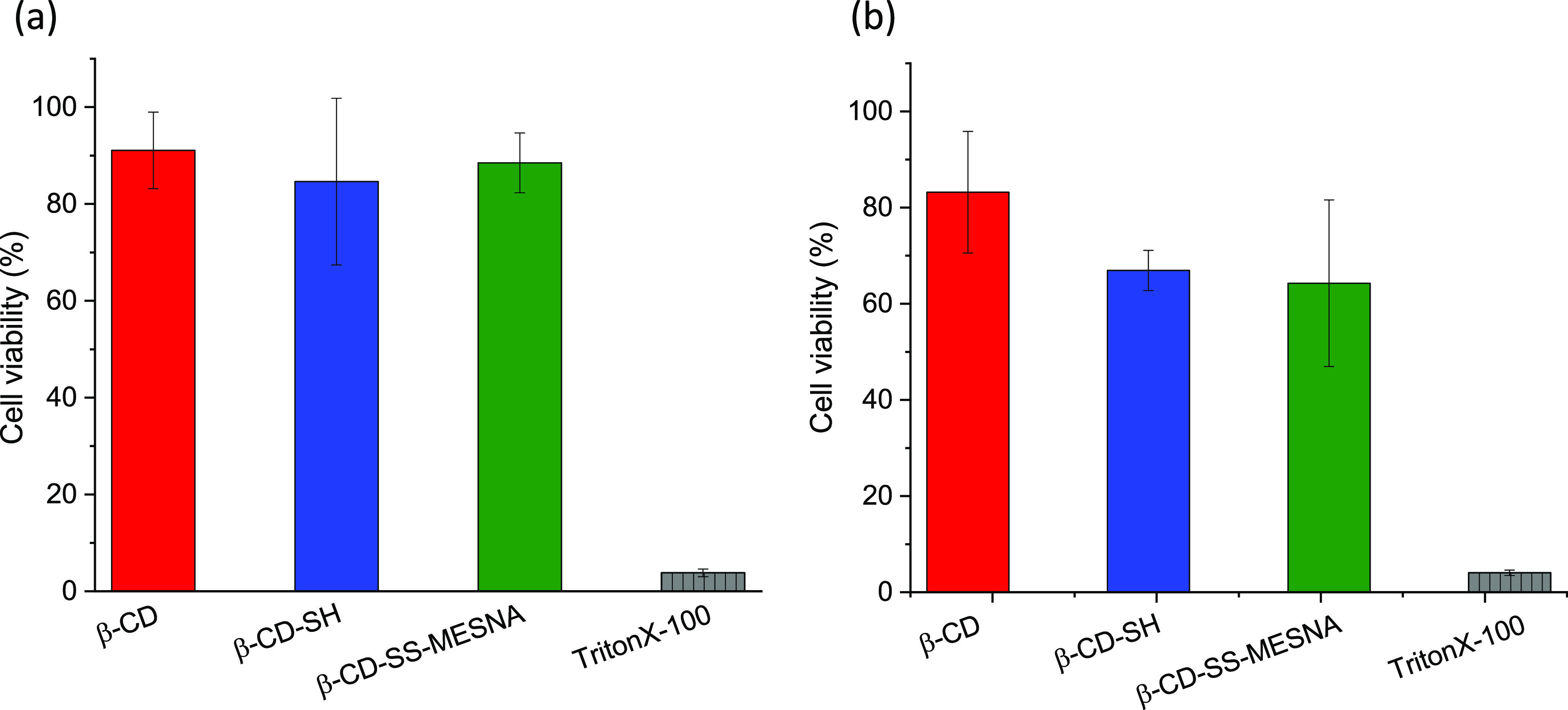
Cell viability of Caco-2 cells after (a) 4 and (b) 24 h of incubation at 37 °C with the native β-CD (red bars), β-CD-SH (blue bars), and β-CD-SS-MESNA (green bars) at 0.25% (m/V) and 0.15% (m/v) Triton X-100 (gray bar) using resazurin assay. Indicated values are illustrated as means ± SD (*n* = 3).

Native and modified β-CDs can extract cholesterol and other lipids from the cellular membrane, leading to cytotoxicity, but to a different extent for various derivatives.^30,31^ Therefore, cell viability studies in the presence of native β-CD, β-CD-SH, and β-CD-SS-MESNA were carried out employing a resazurin assay on Caco-2 cells. This measurement is based on the cellular metabolism of resazurin in living cells. Solutions at a concentration of 0.25% (m/v) were tested for all of the CDs, and the cells were incubated with these solutions for 4 and 24 h. Figure 2 shows only low cytotoxicity of all of investigated solutions, without significant differences within 4 h. After 24 h of incubation, a slightly lower cell viability was found for all CDs.

**Figure 3:**
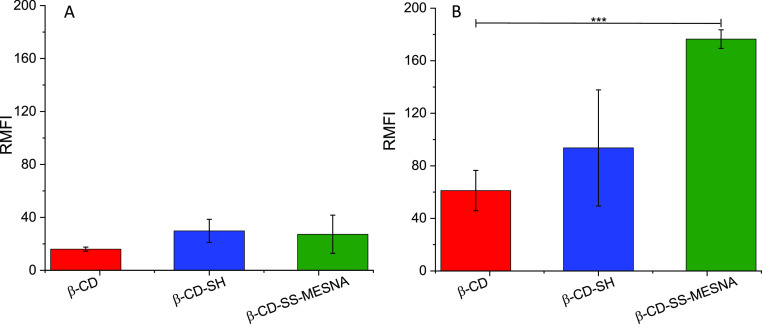
Cellular uptake of indicated CDs; RMFI values of coumarin-6 complexed in the β-CD (red bars), β-CD-SH (blue bars), and β-CD-SS-MESNA (green bars) are presented for Caco-2 cells, measured at 4 °C (A) and 37 °C (B). The data are shown as mean ± SD (*n* = 3) (***P < 0.001).

The interaction of the β-CD and its derivatives with the cellular membrane also increased the cellular uptake.^32^ Our previous paper demonstrated that thiolation of α-CD enhances the cellular uptake.^33^ In order to better understand the cytotoxicity of the synthesized CDs, the cellular uptake of β-CD, β-CD-SH, and β-CD-SS-MESNA/coumarin-6 complexes was analyzed and quantified by flow cytometry. As shown in Figure 3A, low cellular uptake was found for all CDs at 4 °C without significant difference. In contrast, at 37 °C, the cellular uptake increased for all of the CDs (Figure 3B), likely due to the energy-driven endocytosis pathway.^33^ Significant differences in the cellular uptake of applied CDs were found at this temperature. The RMFI values after 3 h of cell treatment with the various CD/coumarin-6 complexes increased 1.5-fold by the thiolation and almost 3.0-fold after S-protection with MESNA. Previous studies showed a slightly higher, 5-fold cellular uptake enhancement by the thiolation of the native α-CD (RMFI 50). For our products, based on the native β-CD with RMFI 60, the 3-fold increase led to an RMFI value of 180, which is in a similar order of magnitude as the cellular uptake using α-CD-SH.^33^ Even though the mechanism of thiol-mediated cellular uptake of CDs is not explored in detail, it is already known that the free thiol groups, as well as disulfides, enhance the cellular uptake through disulfide bond formation with exofacial thiols of the cell membrane proteins. Among the free and oxidized thiols, disulfides enhance cellular uptake to a greater extent and also facilitate endosomal escape.^34^ Due to the higher cellular uptake of β-CD-SH and β-CD-SS-MESNA, more of these CDs are localized and remain in the intracellular matrix, which is most likely responsible for the slightly higher cytotoxicity.

**Figure 4:**
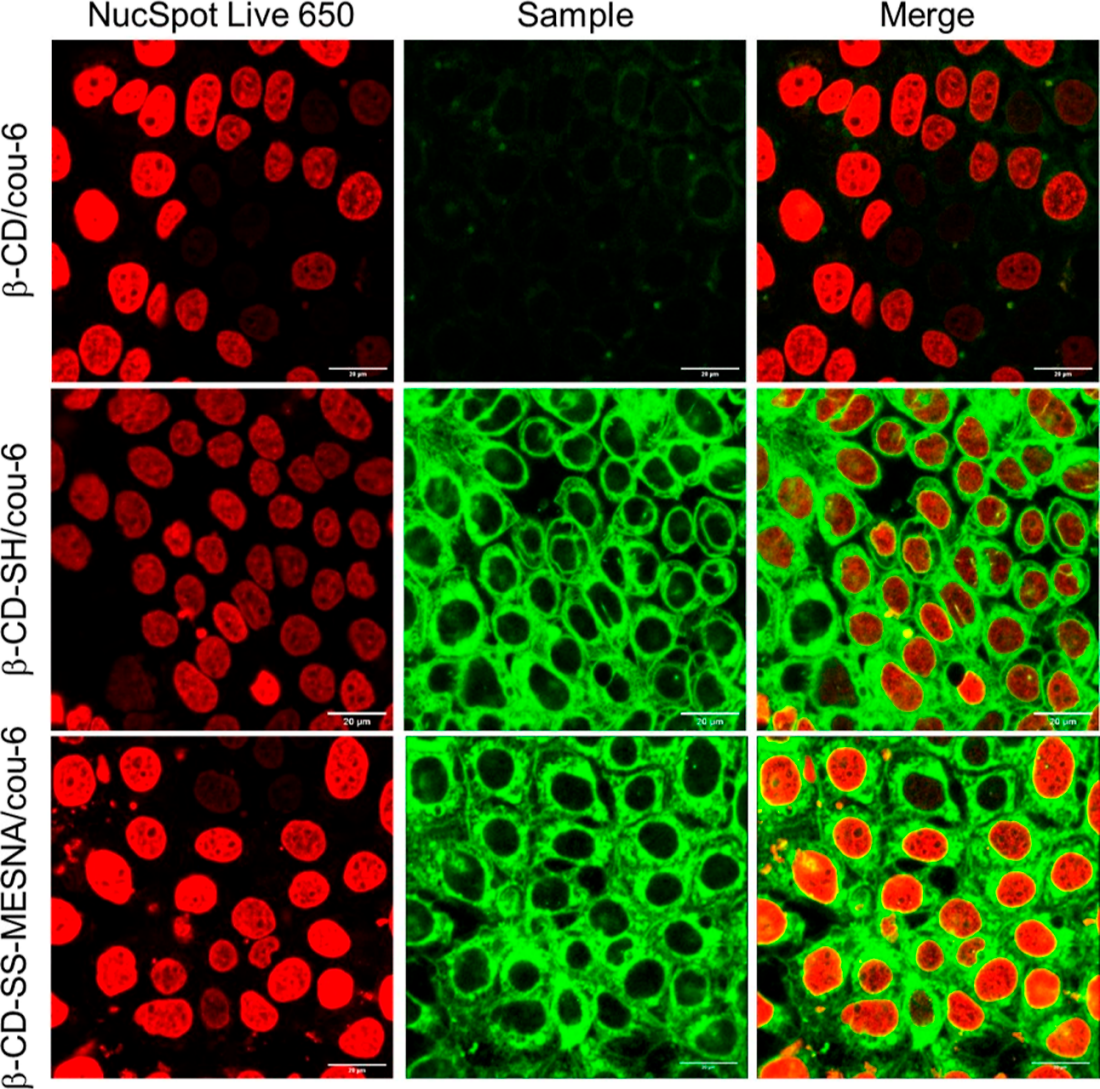
Confocal microscopy images of the uptake of coumarin-6 (cou-6) complexed in β-CD, β-CD-SH, and β-CD-SS-MESNA on Caco-2 cells. The nucleus was stained with NucSpot Live 650.

In order to visualize the cellular uptake, confocal microscopy was used for investigation, and the results are depicted in Figure 4. NucSpot Live 650 nucleic stain was added to the confocal microscopic observations. Therefore, the position of the nuclei is shown by a red signal. Green signals from the fluorescently labeled CD samples showed the amount of internalized CDs. The native β-CD exhibited limited cellular uptake and showed the lowest fluorescence intensity compared to those of thiolated and S-protected thiolated CDs. The lack of specific functional groups hindered its interaction with the cell membrane, resulting in a low internalization. In contrast, β-CD-SH displayed improved cellular uptake compared with the native form. The presence of thiol groups facilitates interactions with the cell membrane, leading to increased internalization, as was expected based on previous studies.^33^ Furthermore, β-CD-SS-MESNA showed further improvement in cellular uptake compared to β-CD-SH. Further investigations are required to elucidate the specific mechanisms and pathways involved in the cellular uptake of β-CD-SS-MESNA.

The two measurement methods gave complementary results, especially in the light that the fluorescence in the confocal microscopy study can also be associated with membrane–bound complex, but flow cytometry measures only the uptaken dye due to the quenched fluorescence in the extracellular volume by trypan blue.^35^

### Mucus Diffusion

**Figure 5:**
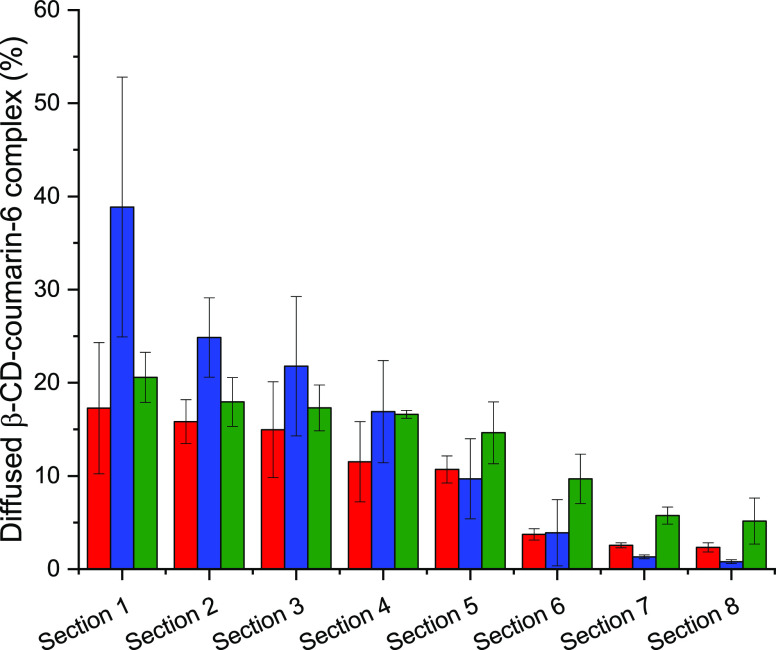
Percentage of coumarin-6, complexed with the β-CD (red bars), β-CD-SH (blue bars), and β-CD-SS-MESNA (green bars) diffused into porcine intestinal mucus after 24 h rotating at 37 °C. Indicated values are means ± standard of at least three experiments.

The diffusion of β-CD, β-CD-SH, and β-CD-SS-MESNA was investigated in silicone tubes filled with mucus. After 24 h of rotation, the diffusivity of these substances was measured. As illustrated in Figure 5, the native β-CD exhibited a higher diffusion rate than β-CD-SH. In fact, the β-CD does not interact with the mucus layer, while β-CD-SH readily forms disulfide bonds with cysteine-rich mucus glycoproteins. These disulfide bonds immobilize β-CD-SH already in the first mucus-filled sections of the tube, to some extent. Therefore, the first section was enriched in β-CD-SH, while lower amounts were found in Sections 2 to 4, and later, the quantity of this product quickly decreased; almost no β-CD-SH was detectable in the last sections. The β-CD-SS-MESNA diffused deeper into the mucus layer compared to β-CD-SH due to the protective effect of the MESNA ligand and was distributed over all of the examined sections. The S-protecting agent assures the resistance against inter- and intramolecular cross-linking, resulting in diffusion even until the last section. The S-protected product reached the last sections of the mucus-filled tube to a greater extent than the native β-CD, most likely due to the anionic MESNA ligand, triggering electrostatic repulsive forces between β-CD-SS-MESNA and the negatively charged mucus gel layer and also because of the mucolytic effect of MESNA, cleaving the mucus glycoproteins on their way to deeper regions. The mucus diffusion enhancing effect was shown previously for other mucoadhesive polymers with anionic substructures^36^ and also by studies describing the mucolytic features of MESNA.^37^ So far, the mucus permeating properties of S-protected thiolated CDs were lower than that of the corresponding native CD.^18,25,38^ Merely, PEG S-protected γ-CD-SH could reach, in the same experimental setup as described in this study, all mucus sections in a detectable amount due to mucus penetrating properties of PEG.^25^

The advantageous property of S-protected β-CD-SS-MESNA lies in its ability to penetrate the firm mucus layer before it interacts with cysteines, which remain unaffected by the natural mucus turnover process. Contrarily, the poorly diffusing β-CD-SH reacts with the loose outer mucus layer, removed rapidly by this mucus clearance process, reducing this excipient’s residence time. Besides the prolonged residence time, the drug carried by this S-protected thiomer is anchored close to the epithelium and should not overcome the mucus barrier to be absorbed and to reach systemic circulation. This finding aligns with the previous research, which indicates the negligible interaction between the native CD and mucus, permitting almost free diffusion through the mucus layer. Conversely, unprotected thiolated CDs exhibit reactivity with cysteine subunits of mucin.^39^ Lam et al. and Knoll et al. conducted studies that support these observations, demonstrating that S-protected thiolated polymers exhibit a more pronounced penetration profile in mucus. This is attributed to a decelerated disulfide bond formation with cysteine-rich subdomains of mucus glycoproteins correlated with polymers with free thiol functionalities.^24,40^ Consequently, the lower reactivity of S-protected thiolated CDs toward intra- and intermolecular cross-linking supports their deeper diffusion into the mucus.

**Figure 6:**
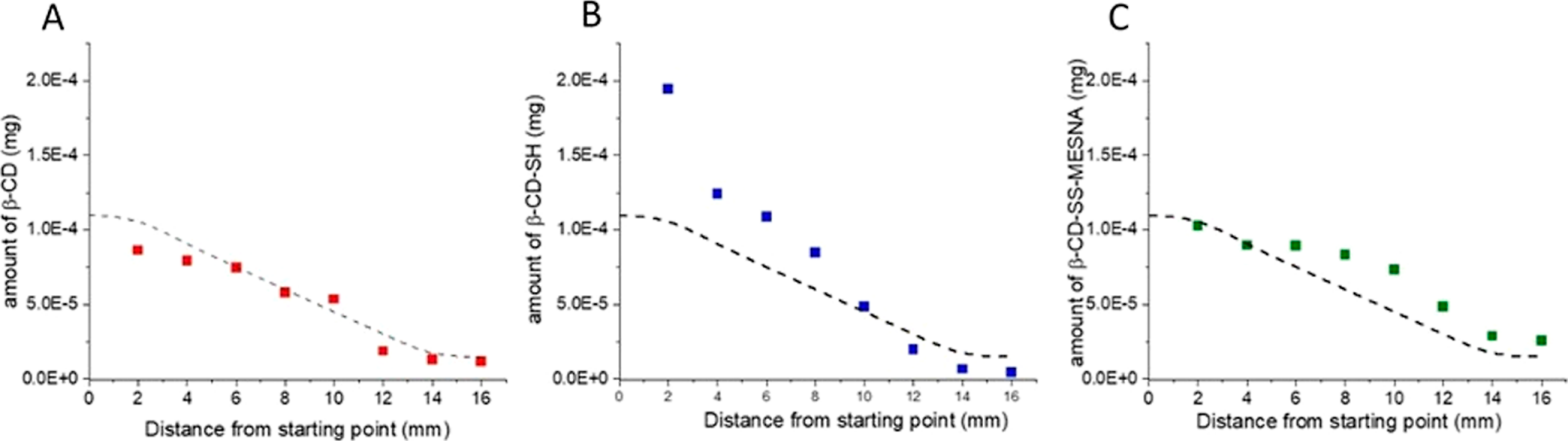
Diffusion of coumarin-6 labeled β-CDs of this study in porcine mucus. Symbols represent the diffused amounts of (A) β-CD (red), (B) β-CD-SH (blue), and (C) β-CD-SS-MESNA (green) after 24 h incubation. The dashed lines indicate the theoretical diffusion of the β-CD with *D*_F_ = 3.78 × 10^–7^ cm^2^/s.

By investigating the amount of diffused CDs as a function of position, the data set can be compared with the diffusion profile of various β-CD derivatives in mucus, as described in the literature.^41,42^ As depicted in Figure 6A, the diffusion profile of the native β-CD correlates with the solution of the free Fickian one-dimensional diffusion model using the diffusion coefficient of *D*_F_ = 3.76 × 10^–7^ cm^2^/s, which is the mean average of values for β-CD derivatives in mucus determined by ^19^F diffusion NMR (dashed lines).^41,42^ In the case of β-CD-SH, a significant deviation from this model is observed (Figure 6B). The first segments are enriched in this CD because of its immobilization in the mucus via disulfide bond formation. At larger distances, the amount of β-CD-SH fell well short of theoretical values. Finally, the β-CD-SS-MESNA presents diffusion similar to the theoretical model but with some enrichment in the later segments (Figure 6C). This increased diffusion is due to the anionic moieties, while the enhanced amount of this derivative in the middle segments is likely connected to the mucoadhesion. In conclusion, the similarity of the diffusion profiles to the literature data for nonmucoadhesive β-CD derivatives is as follows: native β-CD > β-CD-SS-MESNA ≫ β-CD-SH.

### Rheological Investigations

**Figure 7:**
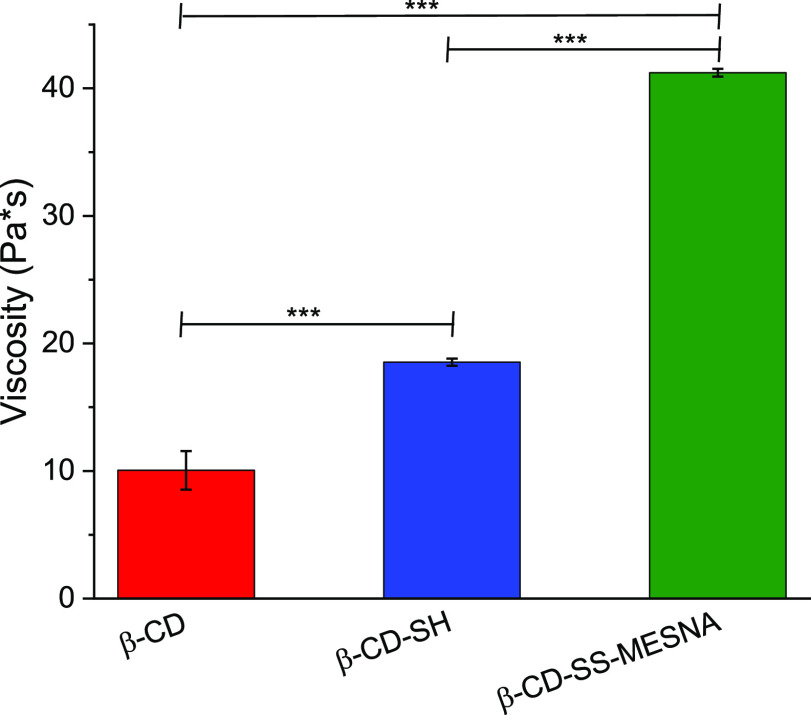
Dynamic viscosity of 0.3% (m/v) β-CD (red bars), β-CD-SH (blue bars), and β-CD-SS-MESNA (green bars) incubated with mucus with a weight ratio of 1:5 at 37 °C for 3 h. Samples were prepared in 0.1 M phosphate buffer pH 6.8. The values are means of at least three experiments ± standard deviation (***P < 0.001).

Due to the thiol/disulfide exchange reaction between the cysteine-rich subdomains of mucus glycoproteins and the thiolated oligomers, new cross-links will be formed with mucus. With an increase in pH, the concentration of thiolate anions increases. These anions are able to form disulfide bonds with other thiol substructures. Measuring the viscosity of the mucus-thiolated CD mixtures at pH 6.8, extensive thiolate anion formation is expected, leading to an elevated number of new disulfide bonds with cysteine substructures, as described previously.^9,23,43^ The disulfides are stable at the pH and temperature applied during measurements. Because of the increase in the cross-linking density, an increased viscosity of the mixture is expected. The interactions between CDs and mucus were studied by evaluating the rheological properties of their mixtures, and the results are depicted in Figure 7. Only a slight, 1.8-fold increase in viscosity was found for the β-CD-SH, compared to native CD. This minor increase in viscosity can be explained by the intermolecular disulfide bond formation between the nonprotected thiol groups of β-CD-SH. After S-protection, this difference was more conspicuous, and in the case of β-CD-SS-MESNA, a significant 4.1-fold increase in viscosity, compared to native β-CD, was detected. This increased mucoadhesion is likely a result of S-protection since protected thiols are less reactive than the free ones, likely penetrating and distributing in the mucus to a greater extent before new disulfide bonds are formed. Generally, PEG, MNA, and MESNA S-protections seem to result in an enhanced mucus viscosity compared to native and thiolated CDs.^25,38^ However, taking the different degrees of thiolation of the S-protected CD described in this study and those from previous studies into consideration, a direct comparison of rheological properties may not be appropriate.

**Figure 8:**
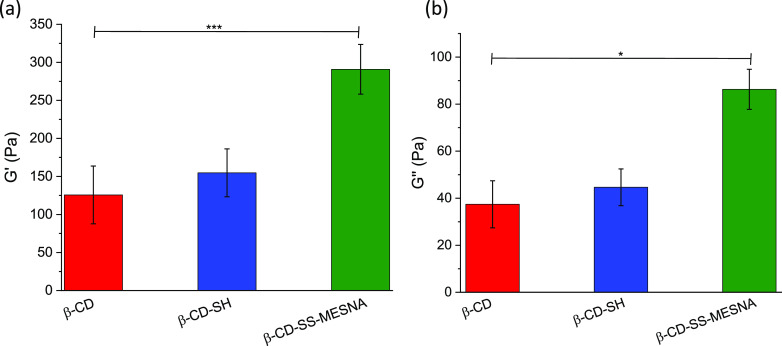
Elastic modulus (*G*′) (a) and viscous modulus (*G*″) (b) of 2% (m/v) β-CD (red bars), β-CD-SH (blue bars), and β-CD-SS-MESNA (green bars) within 3 h of incubation with mucus with a weight ratio of 1:5 at 37 °C and constant frequency 1 Hz. Samples were prepared in 0.1 M phosphate buffer pH 6.8. Indicated values are outlined as means ± SD (*n* ≥ 3) (***P < 0.001, *P < 0.05).

Storage modulus (*G*′) and loss modulus (*G*″) were also determined for all CD mucus mixtures. The same trend was found for β-CD, β-CD-SH, and β-CD-SS-MESNA (Figure 8). A slight, 1.3-fold increase of the *G*′, the elastic component, for β-CD-SH, while a more remarkable 2.3-fold increase for β-CD-SS-MESNA was detected in comparison to the native β-CD. In case of *G*″, the viscous component, the enhancement of viscous behavior was 1.2-fold and 2.3-fold for β-CD-SH and β-CD-SS-MESNA, respectively. These results also confirmed the higher cross-linking density in mucus caused by the disulfide bonds with β-CD-SH or β-CD-SS-MESNA as well as the resistance of the newly formed 3D structure against elastic deformation.

### In Vitro Mucoadhesion Studies on Porcine Small Intestinal Mucosa

**Figure 9:**
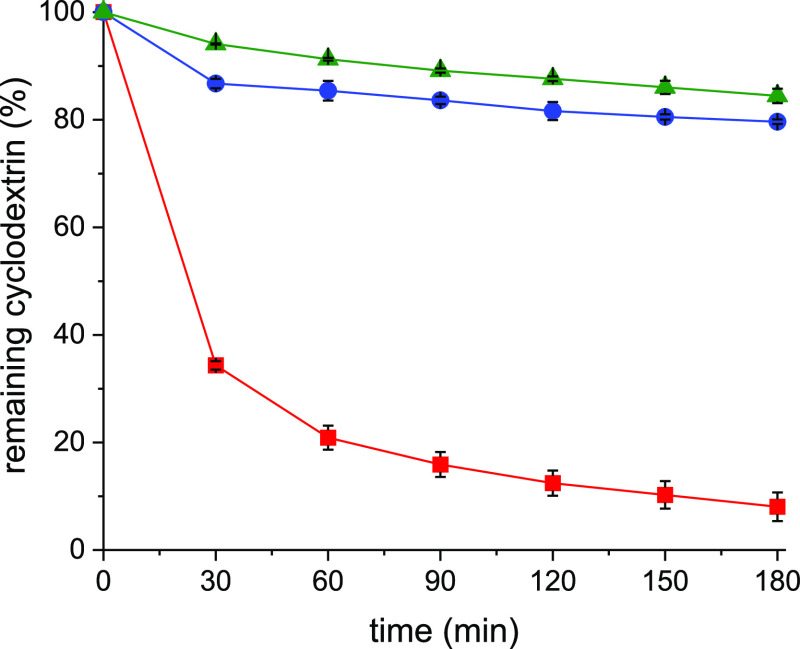
In vitro mucoadhesion of the β-CD (red squares), β-CD-SH (blue circles), and β-CD-SS-MESNA (green triangles) on porcine small intestinal mucosa, rinsed with 0.1 M phosphate buffer pH 6.8 (1 mL/min) at 37 °C. The data are shown as mean ± SD (*n* = 3) (***P < 0.001).

Because of the newly formed disulfide bonds with mucus glycoproteins, a prolonged residence time of thiolated, as well as S-protected, CDs is expected. In order to evaluate the mucoadhesion of native and modified CDs, complexes with coumarin-6 model dye were formed.^10^ The complexes of β-CD, β-CD-SH, and β-CD-SS-MESNA were applied on the freshly excised porcine intestinal mucosa and rinsed with buffer at pH 6.8, and the amount of eradicated complex was determined photometrically. As shown in Figure 9, more than 65% of the native β-CD was washed off in half an hour, and less than 10% remained on the mucosal layer after 3 h. β-CD-SH showed higher mucoadhesion as more than 79% of this compound remained on the mucosal surface within 3 h of continuous rinsing. Finally, almost 85% of β-CD-SS-MESNA was still on the mucosa at the same time point. The 9.8-fold and 10.5-fold increase in mucoadhesion in cases of β-CD-SH and β-CD-SS-MESNA, respectively, compared to parental CD, is provided by the high degree of thiolation as well as S-protection. In line with the previous research,^16^ the S-protected β-CD-SS-MESNA diffused into a deeper mucus region, resulting in a slightly increased mucoadhesion and more prolonged residence time compared to β-CD-SH, which is mostly bound to the loose outer mucus layer that is eliminated by continuous rinsing, similar to intestinal conditions.

Comparing the results obtained in our mucoadhesion studies with data available in the literature for S-protected thiolated CDs, a more prolonged mucosal residence time can be observed. A comparison of these results with literature data, however, has to account that in previous studies α- and γ-CDs were used with different degrees of thiolations.^18,25^

## Conclusions

The highly thiolated β-cyclodextrin (β-CD-SH) was S-protected with MESNA (β-CD-SS-MESNA) in order to reduce oxidative sensitivity and increase mucus diffusion and mucoadhesive properties. The structure of the products was confirmed by ^1^H NMR and FTIR spectroscopy, while Ellman’s test showed highly reduced free thiol content after S-protection. Low cytotoxicity of all the CDs was detected on Caco-2 cell line after 4 h, which increased slightly for β-CD-SH and β-CD-SS-MESNA after 24 h, likely due to the higher cellular uptake of these modified CDs. The β-CD-SS-MESNA exhibited enhanced viscosity and higher elasticity as well as loss modulus after mixing with mucus, compared to the native β-CD and β-CD-SH. S-protection also enhanced the diffusion of the β-CD-SS-MESNA into deep mucus regions, overcoming not only the thiolated but also the native β-CD. The mucoadhesion of the products was enhanced in the following order β-CD < β-CD-SH < β-CD-SS-MESNA. In summary, the decreased reactivity of the thiolated CD by S-protection allows deeper penetration of the carrier into the mucus layer, resulting in stronger mucoadhesion and prolonged residence time, proving our hypothesis. The S-protection also supports cellular uptake, resulting in a marginal increase in cytotoxicity. However, due to the strong mucoadhesion, β-CD-SS-MESNA remains in the mucus layer, slowly eliminated by the mucosal turnover process, not affecting the viability of any cells. Therefore, the S-protection with the MESNA ligand led us to a promising drug delivery excipient for various nonparenteral administration routes.

## Supporting Information Available

400 MHz ^1^H NMR spectra of thiolated and 2-mercaptoethanesulfonic acid S-protected thiolated cyclodextrin and FTIR spectra of the native, thiolated, and 2-mercaptoethanesulfonic acid S-protected thiolated cyclodextrin (PDF)
