# Supplementary material for: Enhanced Mucoadhesion of Thiolated β-Cyclodextrin by S-Protection with 2-Mercaptoethanesulfonic Acid
Source: ACS Omega. 2024 Jan 26;9(5):5819–28. doi: 10.1021/acsomega.3c08836 (PMC10851230; doi:10.1021/acsomega.3c08836)
Supplement: Supplementary file 1 — ao3c08836_si_001.pdf [file ao3c08836_si_001.pdf]

Enhanced mucoadhesion of thiolated β-cyclodextrin by S-protection with 2-mercaptoethanesulfonic acid

Gergely Kali[1], Ali Magdi Mahmoud Mahmoud Taha[1], Emiliano Campanella[1,2], Martyna Truszkowska[1], Soheil Haddadzadegan[1], Nunzio Denora[2], Andreas Bernkop-Schnürch[1]

[1]*Center for Chemistry and Biomedicine, Department of Pharmaceutical Technology, Institute of Pharmacy, University of Innsbruck, Innrain 80-82, A-6020 Innsbruck, Austria*

[2]*Department of Pharmacy, University of Bari Aldo Moro, Piazza Cesare Battisti, I-70121 Bari, Italy*

Corresponding author: Andreas Bernkop-Schnürch, Center for Chemistry and Biomedicine, Department of Pharmaceutical Technology, Institute of Pharmacy, University of Innsbruck, Innrain 80/82, 6020 Innsbruck, Austria. E-mail address: andreas.bernkop@uibk.ac.at

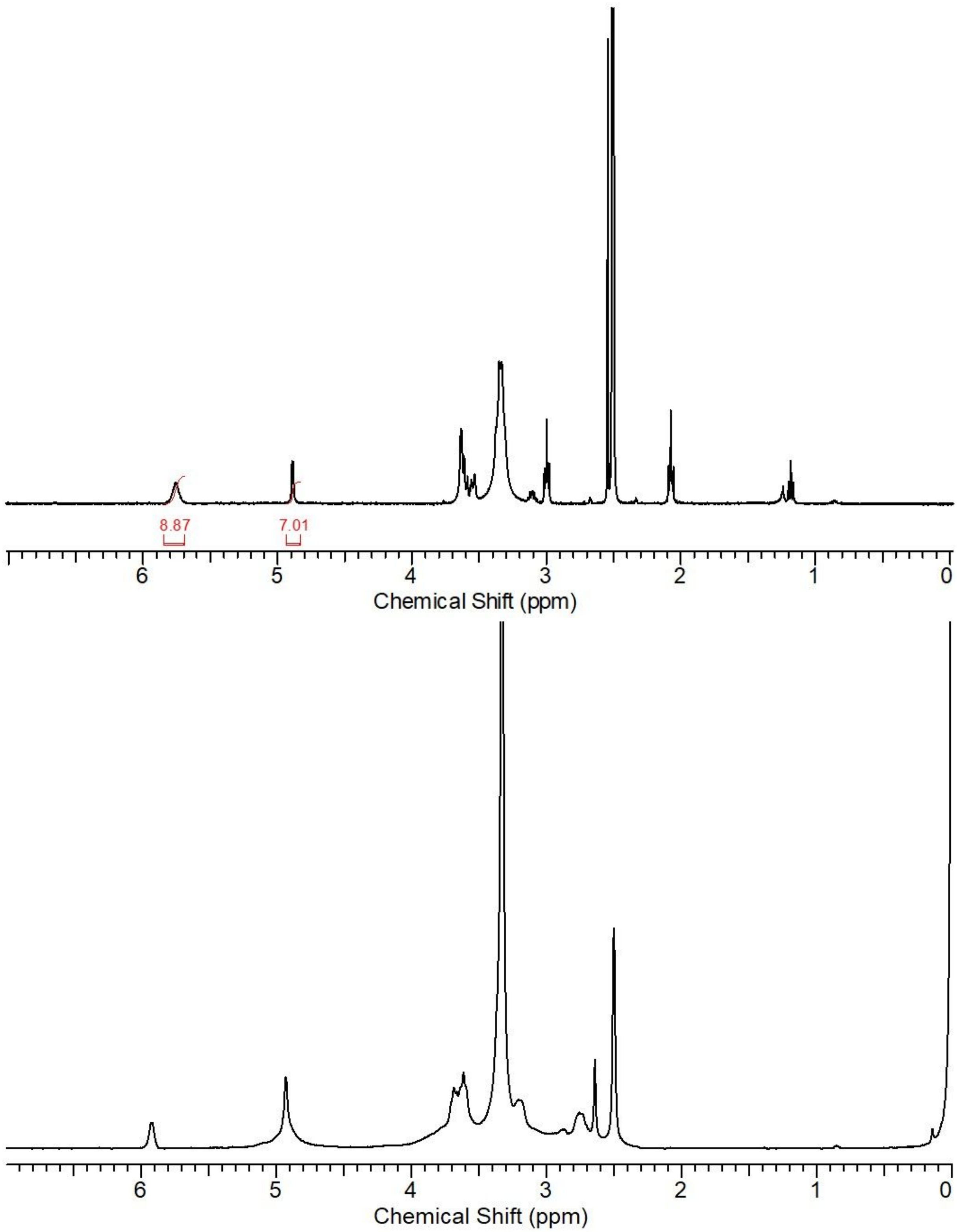

Figure S1. 400 MHz ^{1}H NMR spectra of thiolated (upper spectrum) and 2-mercaptoethanesulfonic acid S-protected thiolated (down-side spectrum) β-cyclodextrin of this work, in DMSO-*d6*.

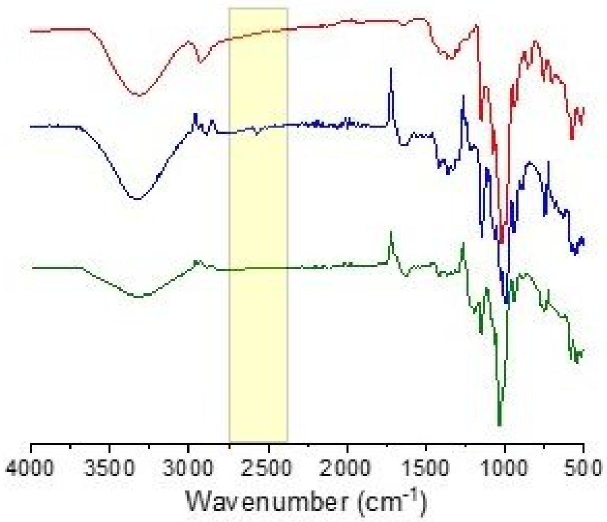

Figure S2. Fourier-transform infrared (FTIR) spectra of the native (red line), thiolated (blue line), and 2-mercaptoethanesulfonic acid S-protected thiolated (green line) β-cyclodextrin of this work.
